# Role of Endogenous and Exogenous Phenolic Compounds on the Formation of Acrylamide and Fluorescent Advanced Glycation End Products in Heated Quinoa and Cañihua Flours

**DOI:** 10.3390/foods15112010

**Published:** 2026-06-04

**Authors:** Ana Aguilar-Galvez, Diego García-Ríos, Teresa Quispe, Cristopher Ames, Andrés Figueroa-Merma, Rosana Chirinos, Franco Pedreschi, Romina Pedreschi, David Campos

**Affiliations:** 1 Instituto de Biotecnología, Universidad Nacional Agraria La Molina, Av. La Molina s/n, Lima 12056, Peru; terequispe902@gmail.com (T.Q.); cristopher.ah@gmail.com (C.A.); 20241785@lamolina.edu.pe (A.F.-M.); chiri@lamolina.edu.pe (R.C.); 2 Research Group on Functional Foods and Nutraceuticals, Universidad Nacional Agraria La Molina, Av. La Molina s/n, Lima 12056, Peru; diego.garcia.r@pucv.cl; 3 Escuela de Agronomía, Pontificia Universidad Católica de Valparaíso, Calle San Francisco s/n, La Palma, Quillota 2260000, Chile; romina.pedreschi@pucv.cl; 4 Departamento de Ingeniería Química y Bioprocesos, Pontificia Universidad Católica de Chile, P.O. Box 306, Santiago 6904411, Chile; fpedreschi@uc.cl; 5 Millennium Institute Center for Genome Regulation, Santiago 8320000, Chile

**Keywords:** andean pseudocereals, thermal processing, maillard reaction, antioxidants, acrylamide, fluorescent AGEs

## Abstract

In this study, the formation of Maillard reaction products (MRPs), namely acrylamide (AA) and fluorescent advanced glycation end products (f-AGEs), was evaluated in four quinoa and four cañihua flours subjected to heating at 185 °C. The influence of endogenous phenolic compounds and the addition of tara hydrolyzed gallotannins on MRPs formation was investigated. Varieties with higher quercetin and rutin contents showed lower AA and f-AGEs formation. AA formation kinetics differed between species, increasing with heating time in quinoa, whilst decreasing after 20 min in cañihua. Maximum AA levels ranged from 18.6 to 87.0 μg/kg, remaining below the EU benchmark value for non-wheat-based bread (300 μg/kg). Compared with the Asn–Glc control system, flour systems reduced AA formation by approximately 18.7 to 60.3%, while tara hydrolyzed extract further enhanced inhibition, reaching reductions above 72.7 to 96.2%. Similar trends were observed for f-AGEs, with mitigation levels up to 54.1 to 77.4%. Cañihua varieties showed higher AA inhibition capacity than quinoa, likely associated with differences in phenolic composition and antioxidant capacity. These findings demonstrate the potential of Andean pseudocereals and tara-derived polyphenols as natural strategies to mitigate thermally induced contaminants in processed foods.

## 1. Introduction

Thermal treatment is a widely used preservation technology for ensuring food safety, shelf-life improvement, and the development of desired sensory characteristics. These processes include high temperature unit operations such as evaporation, pasteurization, drying, sterilization, steaming, boiling, grilling, microwave heating, and baking, among others. Nevertheless, aside from these benefits, heating induces chemical modifications that also lead to the formation of harmful compounds known as neo-formed contaminants (NFCs) [1].

Among the main NFCs, acrylamide (AA), furan, 5-hydroxymethylfurfural (5-HMF), heterocyclic amines (HCAs), and imidazoles are found. In addition, advanced glycation end products (AGEs) are also a diverse and complex group of potentially harmful compounds produced by thermal processing. These compounds are generated by the reactions between amino groups from protein and free amino acids and carbonyl groups from sugars at high temperatures (>120 °C) known as the Maillard reaction (MR) [2,3].

The presence of these harmful compounds in the diet has been associated with adverse health effects, including carcinogenicity, neurotoxicity, mutagenicity, and chronic diseases such as diabetes and cardiovascular pathologies [4,5]. Consequently, the development of strategies for mitigating its occurrence in foods is an important task for the food industry. Among the proposed mitigation techniques, incorporating natural compounds such as polyphenols, vitamins and peptides has demonstrated a promising potential for inhibiting the MR and thus reduce NFCs formation [6,7].

Andean pseudocereals such as quinoa (*Chenopodium quinoa* Willdenow) and cañihua (*Chenopodium pallidicaule* Aellen) represent promissory sources of both nutrients and bioactive compounds. Quinoa stands out for its unsaturated fatty acid profile, minerals, vitamins, phenolic acids and flavonoids [8,9]. Cañihua is rich in protein, essential amino acids, dietary fiber, and has lower saponin levels than quinoa [10].

While there is increasing evidence of the role of polyphenols in inhibiting NFCs formation in foods, there is still a knowledge gap regarding Andean pseudocereals and the potential of their endogenous polyphenol contents as NFCs inhibitors. In the case of AA mitigation, many mechanisms have been proposed for the inhibiting effect of polyphenols such as (i) trapping of highly reactive carbonyl and dicarbonyl compounds which are essential for initiation of the MR, (ii) prevention of lipid oxidation, which also reduces the amount of reactive aldehydes produced by fatty acid decomposition, (iii) interfering in the conversion of key intermediates such as 3-aminopropionamide to AA, and (iv) neutralizing other reactive intermediates through formation of covalent bonds that block their capacity to participate in the reaction route [11]. In addition, the magnitude of NFC formation is largely determined by the matrix composition with respect to Maillard reaction precursors such as reducing sugars and free amino acids [12,13]. Thus, both polyphenols and Maillard reaction precursors should be evaluated under controlled heating conditions to understand NFCs formation in quinoa and cañihua.

In addition, exogenous polyphenol sources have also been considered for NFCs mitigation in foods. In particular, tara pods (*Caesalpinia spinosa*) are a rich source of tannins and other polyphenols with recognized antioxidant activity [14] and with the ability to quench intermediate products of the MR and thus mitigating AA formation in starchy food matrices [15,16]. The addition of antioxidant extracts from tara would reduce the formation of AA and AGEs in Andean pseudocereals while increasing their functional value.

Despite the increasing evidence about the effects of natural polyphenols as inhibitors of the formation of NFCs, there are still some relevant knowledge gaps. Thus far, studies have focused on conventional food matrices and pure phenolic compounds. Meanwhile, available information about the role of endogenous quinoa and cañihua polyphenols on mitigating NFCs formation during thermal processing is limited. In particular, the relationship between MR precursors, phenolic compound profile and the simultaneous formation of AA, f-AGEs and other MRPs on these pseudocereals has not been completely elucidated. Moreover, evaluations encompassing different quinoa and cañihua varieties as well as the complementary effect of exogenous polyphenols (such as tara pod gallotannins) are still limited.

This study poses that endogenous polyphenol composition of quinoa and cañihua flours and its supplementation with tara pod polyphenols modulates the Maillard reaction and thus is able to reduce NFCs formation during thermal treatment. Therefore, the aim of this study was to (i) evaluate the profile of precursors and potential inhibitory compounds related to NFCs formation in four varieties of quinoa and cañihua flours, (ii) assess the formation of NFCs, and other MRPs under thermal treatment at 185 °C for 20, 30 and 40 min (chosen such that typical temperatures and times of culinary and industrial processes are represented) and relate it to their endogenous polyphenolic profile, and (iii) determine the effect of tara polyphenol addition on NFCs, in a glucose–asparagine model system containing quinoa or cañihua flour.

## 2. Materials and Methods

### 2.1. Plant Materials

Grains from four varieties of quinoa (*Chenopodium quinoa*)—“Cuchiwilla”, “Chullpy”, “Salcedo INIA”, and “Negra Collana”—and four varieties of cañihua (*Chenopodium pallidicaule*)—“Rojo Ramillete”, “Gris Alfenica”, “Amarillo Chilligua” and “Illpa INIA”—were acquired from Puno, Peru (Supplementary Figure S1). For each variety, three independent processing batches (n = 3) were prepared, each consisting of 200 g of grains used for flour production. The resulting flours were used for all subsequent analyses. This experimental design ensured technical replication of the processing and analytical procedures for each variety. The total grain amount per variety ranged from 3.8 to 4.6 kg for quinoa and from 3.6 to 4.1 kg for cañihua. Tara pod (*Caesalpinia spinosa*) flour was obtained from a local supplier in Lima, Peru. Tara pod extract was obtained according to the procedure described by Campos et al. [15], then freeze-dried and stored in airtight polyethylene bags until use.

### 2.2. Reagents and Chemicals

DMF (1-deoxy-1-morpholino-d-fructose), ABTS (2,2′-azino-bis(3-ethylbenzothiazoline-6-sulfonic acid), DNS (3,5-dinitrosalicylic acid), Trolox (6-hydroxy-2,5,6,8-tetraethylchroman-2-carboxylic acid), and AAPH (2,2′-azino-bis(2-methylpropionamide) dihydrochloride) were purchased from Sigma-Aldrich (St. Louis, MO, USA). Analytical standards of acrylamide (23701), d3-acrylamide (72334), l-asparagine (A0884), and Nα-acetyl-l-lysine (A2010) were purchased from Sigma-Aldrich (Buchs, Switzerland). Proteases: Pepsin from porcine gastric mucosa (P7012), Trypsin from porcine pancreas, type II (T7409), α-Chymotrypsin from bovine pancreas, type II (C4129) were purchased from Sigma (St. Louis, MO, USA). All other reagents and solvents were of analytical grade or MS grade and were obtained from Merck (Seelze, Germany), J.T. Baker (Phillipsburg, NJ, USA) and, LiChrosolv^®^ (Darmstadt, Germany), respectively.

### 2.3. Flour Preparation and Thermal Treatment Conditions

Quinoa and cañihua grains were manually selected to remove impurities. Then, they were washed with tap water (grain: water, 1:5, *p*/*p*), let drip for 5 min and dried in a drying oven (Venticell 22, MMM Medcenter, Planegg, Germany) at 50 °C with a constant air flow until moisture less or equal to 10% was reached. Upon drying, the grains were ground in a laboratory mill (PULVERISETTE 14, FRITSCH^®^, Idar-Oberstein, Germany) and passed through a 500 µm sieving mesh (35 mesh). The obtained flours were packed in airtight polyethylene bags and stored at −20 °C until use.

The thermal treatment assay was performed according to the previously described methodology [17] with slight modifications. Firstly, the flours were conditioned in a closed system containing a saturated KOH solution at 10 °C for 24 h to equilibrate the moisture to around 6%. Afterwards, a container filled with sand was preheated to 185 °C (average baking temperature for biscuits), and a glass tube containing 2.2 g of sample was subsequently placed inside the sand bath for 20, 30 and 40 min. Following the treatment, the sample was rapidly cooled in cold water (~4 °C) to stop the thermally induced reactions.

### 2.4. Model System Assay

A glucose–asparagine (Glc-Asn) model system was prepared according to the method described by Cheng et al. [18] with some modifications. Three hundred mg of flour (whose contents of Glc and Asn were previously determined) were weighed on glass tubes. Then, pure Glc and Asn were added such that an equimolar concentration of 0.1 M was reached. Tara pod hydrolyzed extract (THE, 99.6% gallic acid of total gallotannins) was added to that mixture at two concentrations (5 and 10 mg GAE/mL). A control sample without THE addition and a positive control consisting of pure Glc-Asn were also assayed. All samples were diluted to a final volume of 6 mL by addition of phosphate buffer (10 mM, pH 7.4). After mixing by constant stirring for 30 min, the tubes were heated at 185 °C for 20 min as described in Section 2.3. After cooling down, the tubes were centrifuged at 13,751× *g* for 12 min at 20 °C. The supernatant was collected for further analysis.

### 2.5. Analytical Methods

#### 2.5.1. Sugars, Starch, and Asparagine Determination

The extraction and determination of sugars (glucose, fructose, and sucrose), reducing sugars, and asparagine were carried out according to Campos et al. [15]. Starch determination was performed according to AOAC method 996.11 [19]. Sugars, reducing sugars and starch were expressed as g/100 g DW whereas asparagine was expressed as mg/100 g DW.

#### 2.5.2. Total and Soluble Protein

Total protein in flour samples was determined by the Kjeldahl method [19] (conversion factor of 5.85). Soluble protein was extracted with a 0.9 M NaCl solution and then determined by the methodology reported by George & Christoffersen [20]. Briefly, 10 µL of the obtained extract and 200 µL of Bradford reagent (Bio-Rad Laboratories, Hercules, CA, USA) were mixed in a 96-well microplate and after 10 min the absorbance at 595 nm was measured. A standard calibration curve was constructed with bovine serum albumin (BSA) protein in the concentration range of 0.05 to 0.5 mg/mL.

#### 2.5.3. Free Amino Group

The determination of the free amino group on flour samples after heating was performed according to Michalska et al. [21]. Briefly, samples were mixed with 6% sodium dodecyl sulfate such that the total protein content was 6 mg in 3 mL of reaction volume. Then, they were mixed by agitation for 10 s every 10 min three times. After that, they were centrifuged at 13,751× *g* for 5 min at room temperature and the upper phase was filtrated through Whatman N° 40 paper. The filtrate (50 µL) was placed in a 96-well plate with 100 µL of distilled water, 100 µL of OPA reagent, and mixed for 3 min. Fluorescence (λ_Ex_ = 340 nm y λ_Em_ = 455 nm) was measured using a FLUOstar Omega microplate reader (BMG LABTECH GmbH, Ortenberg, Germany). A standard curve of Nα–acetil-l-lysine was constructed in the 50–500 µM concentration range, and results were expressed as g Nα–acetil-l-lysine per 100 g of total protein.

#### 2.5.4. Total Phenolic Compounds, Phenolic Profile and In Vitro Antioxidant Capacity Determination

Total phenolic compounds (TPC) and antioxidant capacity by ABTS (AC-ABTS) and ORAC (AC-ORAC) were performed following the previously described methodology [22]. TPC were expressed as mg gallic acid equivalent (mg GAE/g DW) and the antioxidant capacity in both methods was expressed in micromole of Trolox equivalents (µmol TE/g DW). The phenolic profile of the quinoa and cañihua varieties was carried out by UPLC-PDA-ESI-QToF-MS^e^ (ACQUITY UPLC I Class, Waters Corp., Milford, MA, USA) as previously described [22].

#### 2.5.5. Maillard Reaction Products (MRPs): Early MRPs, Advanced MRPs, and Brown Pigments

Early MPRs (Maillard reaction products) were determined by nitroblue tetrazolium (NBT) assay that quantifies fructosamine as Maillard reaction progression marker [23]. A fructosamine analog DMF (1-deoxy-1-morpholinofructose) was used to build a calibration curve. Advanced MRPs and brown pigments were extracted by enzymatic digestion and determined following the procedure described by Borrelli et al. [24]. Advanced MRPs were detected by fluorescence (λ_Ex_ = 360 ± 40 nm and λ_Em_ = 460 ± 40 nm). Brown pigments were determined by measuring absorbance at 420 nm. All results were expressed on a total protein content basis.

#### 2.5.6. Acrylamide Determination

Acrylamide (AA) analysis was carried out following the methods described by Bertuzzi et al. [25] and Campos et al. [15] with some modifications. For the flours, 0.5 g were weighed on a centrifuge tube and mixed with 1.25 mL of hexane, 2.5 mL of ultrapure water, 2.5 mL of acetonitrile, and 20 µL d3-acrylamide (1 µg/mL) as internal standard. For the model system (Asn-Glc), an aliquot of 125 µL of the system supernatant was mixed with 500 µL of phosphate buffer, 500 µL of hexane, 1000 µL of acetonitrile and d3-acrylamide. After mixing vigorously for 1 min, anhydrous magnesium sulphate and sodium chloride were added and mixed for 5 min. Then, the samples were centrifuged at 8800× *g* for 10 min at 15 °C and the upper phase (acetonitrile) recovered. The residue was extracted once more with 2 mL of acetonitrile. An aliquot (1.5 mL) of the extract was further cleaned-up using QuEChERS (KS0-9514, Phenomenex^®^, Torrance, CA, USA) and after centrifugation, 800 µL of the upper phase were dried under a nitrogen stream and reconstituted with 160 µL of ultrapure water and filtered through a 0.22 μm membrane. Chromatographic analysis was performed as previously described [15] using a HSS T3 column (100 mm × 2.1 mm i.d., 1.8 µm particle size; Waters, Dublin, Ireland). The mobile phase consisted of ultra-pure water with 0.5% methanol and 0.05% formic acid delivered at a flow rate of 0.2 mL/min and 30 °C in isocratic mode. In total, 2 µL of the cleaned-up extract were injected and separation was achieved in 7 min. Detection was performed on a TSQ Quantum Access Max triple quadrupole mass spectrometer (Thermo Scientific, San Jose, CA, USA). Positive polarity electrospray ionization (ESI) was performed with 3.5 kV ionization voltage, 250 °C vaporizer temperature, 270 °C capillary temperature, sheath gas pressure of 20 (arbitrary units) and auxiliar gas pressure of 5 (arbitrary units). Single reaction monitoring (SRM) method was used for detection of AA (m/z 72.1 > 53.3) and d3-AA (75.1 > 58.3) transitions used for measurement of peak chromatographic areas. Data acquisition was performed using Chromeleon™ (version 6.80 SR15b) and TSQ Tune™ (version 2.5.0.1307), and the resulting data were processed using the Xcalibur™ (version 3.0.63) (all software from Thermo Fisher Scientific Inc., Waltham, MA, USA). Quantification was performed by constructing a calibration curve using AA and d3-AA analytical standards.

#### 2.5.7. Fluorescent Advanced Glycation End Products

The determination of fluorescent advanced glycation end products (f-AGEs) was performed according to Zhu et al. [17]. For thermally treated flours, 0.5 g were weighed and mixed with 2.5 mL of distilled water. Then, the mixture was placed in an ultrasonic bath at 40 °C for 10 min. Lastly, it was centrifuged at 13,751× *g* for 15 min at 4 °C and the upper phase recovered. Fluorescence was measured on 96-well plates (Brand, 781605, Germany); 150 μL of sample were placed in the microplate well and fluorescence was measured using the same parameters as indicated in Section 2.5.5 (λ_Ex_ = 360 ± 40 nm and λ_Em_ = 460 ± 40 nm). The results were expressed as fluorescence units (FU) per g DW in the case of flours and mitigation percentage with respect to the positive control for the model systems.

### 2.6. Statistical Analysis

All results are presented as the mean of three repetitions with their standard deviation. One-way analysis of variance (ANOVA) followed by Tukey’s post hoc test was used (*p* < 0.05) for variety comparison for each species tested, using the Statgraphics Centurion XVI (StatPoint Inc., Rockville, MD, USA). Principal component analysis was performed using The Unscrambler^®^ X version 10.4 (CAMO Software, Oslo, Norway) to explore the relationship between Maillard reaction markers and the formation of AA and f-AGEs with heating time.

## 3. Results and Discussion

### 3.1. Maillard Reaction Precursors and Potential Inhibitors Content and Profile

The main Maillard reaction precursors (asparagine, glucose, and sucrose), total phenolics and antioxidant capacity are shown in Table 1. Significant differences among varieties were observed. In both grain species, sucrose was found in higher concentrations than glucose, which is consistent with a pattern reported for quinoa grains by Pellegrini et al. [26]. Interestingly, no fructose was found in any variety but in cañihua “Amarillo Chilligua” (0.04 g/100 g DW). Asparagine, a key AA precursor, also presented varietal variability in both species, suggesting a higher AA formation potential for quinoa “Cuchiwilla” and cañihua “Rojo Ramillete”.

With respect to compounds with potential capacity to inhibit MRPs such as phenolic compounds, cañihua showed higher TPC concentrations than quinoa. Interestingly, colored varieties for both species presented higher TPC levels, a trend which was previously observed for other quinoa varieties [27]. Although higher TPC values have been previously reported for quinoa (between 4.4 and 6.4 mg GAE/g DW) [28], the pattern of phenolic abundance (black > red > white) holds for the samples analyzed in this study. In the case of cañihua, a high TPC concentration range (2.1–12.1 mg GAE/g DW) has been previously reported for Bolivian ecotypes [29], highlighting the great influence of genetics and cultivation conditions on the phenolic and other bioactive concentrations [30]. In general, α-carbonyl trapping capacity has been attributed to phenolic compounds, and blocking these highly reactive compounds such as GO and MGO could hinder the Maillard reaction progression, reducing NFCs and AGEs formation [31].

Regarding antioxidant capacity, quinoa “Negra Collana” and cañihua “Amarillo Chilligua” presented the highest AC-ABTS whereas quinoa “Salcedo INIA” and cañihua “Rojo Ramillete” had the greatest AC-ORAC. For quinoa, the values were higher than previously reported for white, red and black varieties (4.5–5.4 µmol TE/g DW, ABTS and 4.8–5.4 µmol TE/g DW, ORAC) [32]. In the case of Bolivian ecotypes, a lower AC-ABTS antioxidant capacity range was reported (1.8–7.8 µmol TE/g DW) [29].

### 3.2. Phenolic Compounds Profile in Quinoa and Cañihua Flours

Given the structure-dependance of phenolic compounds on their activities, a further characterization of these compounds by LC-PDA-QToF-MS^e^ was performed. Supplementary Tables S1 and S2 show the profile and tentative annotation for quinoa and cañihua phenolics, respectively. In both species, flavonoids were the main phenolic group, representing over 90% of the total phenolics. Quercetin glucoside derivatives, characterized by the 255 ± 1 nm and 355 ± 1 nm absorption peaks and the m/z 300 ± 1 Da fragment, were predominant in all quinoa varieties. Rutin, a well know quercetin glucoside was only found in quinoa “Chuchiwilla”. In contrast, rutin was detected in all cañihua varieties, along with quercetin glucoside derivatives. Previous works have also found that flavonoids (quercetin and kaempferol derivatives) are the main phenolic compounds in pseudocereals [33].

### 3.3. Effect of Thermal Treatment on Maillard Reaction Kinetics

Maillard reaction is generally thought to have three main stages: early, advanced, and final [21]. To better understand the effects of the compositional diversity on the formation of MRPs (AA, f-AGEs), markers of each MR stage have been evaluated in this study.

**Figure 1:**
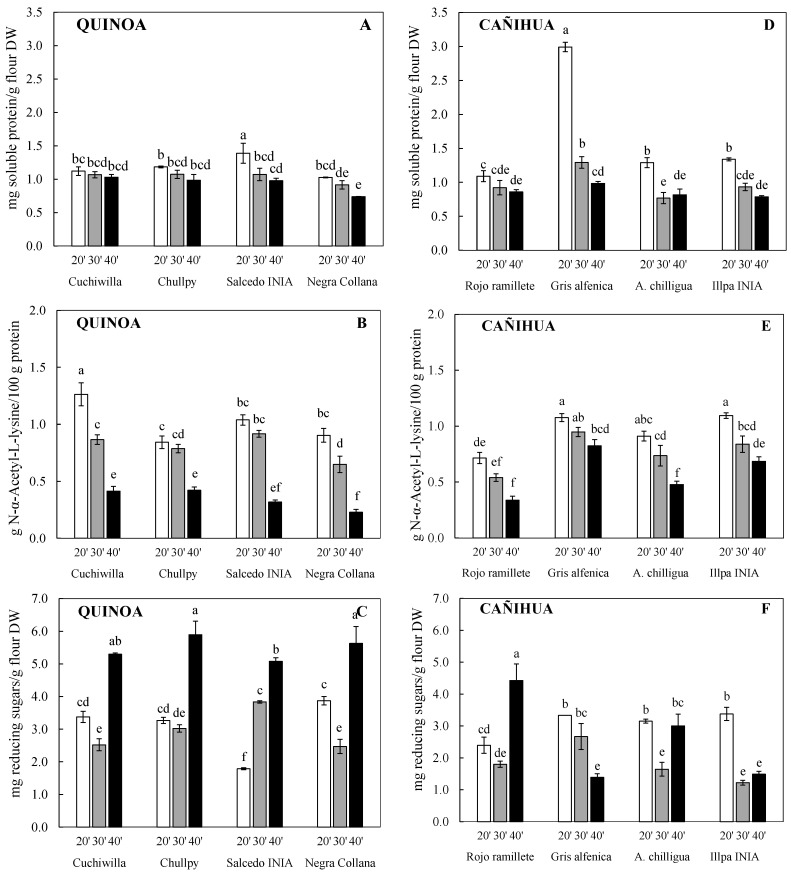
Maillard reaction precursors. Soluble protein (**A**,**D**), free amino groups (**B**,**E**), reducing sugars (**C**,**F**). Data are presented as means ± SD (n = 3). Different lowercase letters indicate significant differences (*p* < 0.05) determined by a two-way ANOVA (variety × heating time).

Figure 1 shows the evolution of soluble protein, free amino groups (proxy for available lysine) and reducing sugars in quinoa and cañihua flours subjected to heating at 185 °C for 20, 30, and 40 min. In all varieties for both species, soluble protein decreased progressively with heating time (Figure 1A,D), a result in agreement with previous studies [34,35] that remark the interaction between soluble proteins and reducing sugars with MRP formation, browning, increase in antioxidant activity and sensory properties development of thermally treated foods. Thus, the loss of soluble protein indicates the progression of the initial phase of the MR. Free amino groups (Figure 1B,E) also decreased with heating time, suggesting their interaction with reducing sugars during heating. These obtained values for free amino groups were half of those reported for rye flour [21]. Interestingly, in both wheat [23] and rye breads, lower free amino groups were detected in the crust than in the crumb, suggesting a higher degree of lysine blocking due to the Maillard reaction and to the lower moisture content of the crust conditions that are similar to those experienced by the pseudocereal flours used in this study. These results suggest that pseudocereals could have a different glycation dynamic than traditional cereals, although this is dependent on the preparation method (flour, bread, or other bakery products). In contrast, for reducing sugars (Figure 1C,F) the reduction with heating was not consistent, with some varieties showing reductions from 20 to 30 min and then an unexpected increase after 40 min of heating. An increase in reducing sugars with heating was observed in quinoa “Salcedo INIA”, whereas only in cañihua “Gris Alfenica” and “Illpa INIA” a reduction with heating was noted. A reduction of reducing sugars with heating is expected due to their condensation with free amino groups to form unstable Schiff bases [4,36]. The observed increases could be attributed to the thermal degradation of other sugars such as sucrose or even starch that could contribute to the reducing sugars pool during prolonged heating [37]. The concomitant decreases in soluble protein, free amino groups, and reducing sugars suggest their participation in thermal-induced reactions and Maillard reaction products formation.

**Figure 2:**
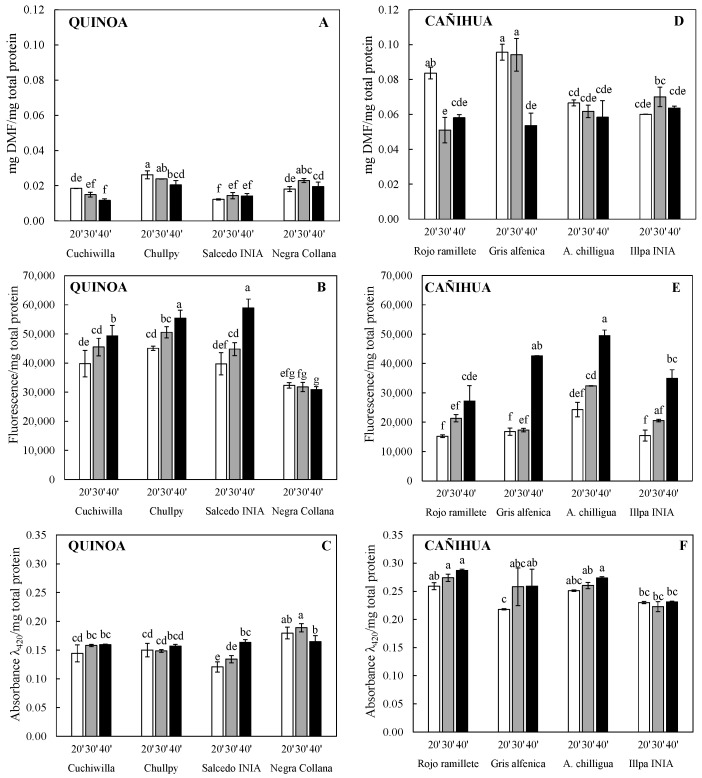
Maillard reaction progression markers: early (**A**,**D**), advanced (**B**,**E**), and final (**C**,**F**). Data are presented as means ± SD (n = 3). Different lowercase letters indicate significant differences (*p* < 0.05) determined by a two-way ANOVA (variety × heating time).

Regarding markers of the progression of the MR, fructosamine (DMF), a key early MR intermediate [38], was found in higher concentrations in cañihua than in quinoa heated flours (Figure 2A,D). The decrease in DMF could be attributed to its participation as a substrate for advanced glycation reactions [39,40]. Fluorescent MRPs, related with intermediate–advanced stages of the MR, showed an increasing trend with heating (Figure 2B,E), which agrees with previous observations by Silvan et al. [40]. Only in quinoa “Negra Collana” were no significant differences (*p* > 0.05) found between fluorescence and heating time. Brown pigments are related to the late or final stage of the MR, signaling the accumulation of melanoidins and other polymers [41]. A slight increase was observed with heating time, though in some varieties no significant increase was observed (Figure 2C,F). Melanoidins could originate from both MR and oxidation and polymerization of phenolic compounds [23] and fructosamine condensation [42].

### 3.4. Acrylamide and Fluorescent AGEs Formation During Heating

**Figure 3:**
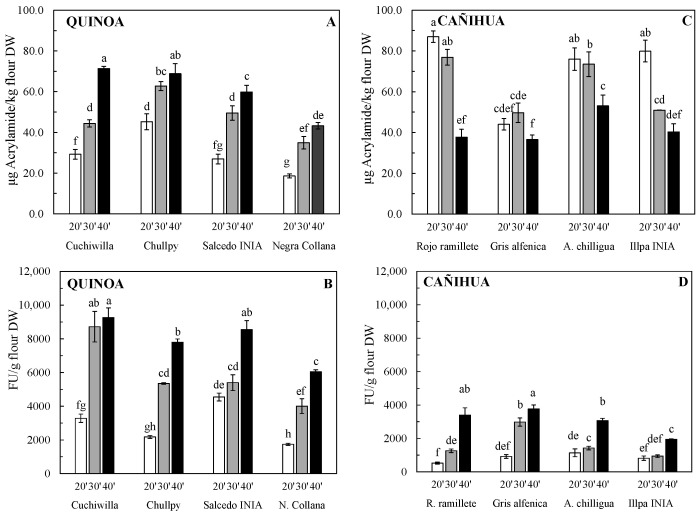
Acrylamide (**A**,**C**) and fluorescent AGEs (**B**,**D**) formation with heating time in quinoa and cañihua flours. Data are presented as means ± SD (n = 3). Different lowercase letters indicate significant differences (*p* < 0.05) determined by a two-way ANOVA (variety × heating time).

Figure 3 shows the different formation kinetics of AA in quinoa and cañihua flours produced by heating (Figure 3A,C). Quinoa was characterized by an increase in AA contents with heating time, reaching the highest values at 40 min of the treatment. Conversely, cañihua AA values peaked after 20 min of heating, followed by a decline as heating progressed. These differences signal the complex relationship between precursors, inhibitors and intermediate products of the MR, and that varietal differences should be considered to optimize thermal processing to mitigate this toxic compound [43]. Notably, these observed AA values were lower than those reported for wheat and rye flours treated at 160–180 °C for 20 min (2000–6000 µg/kg) [44]. High AA values have also been reported for other cereals, namely sorghum (160 µg/kg), millet (446 µg/kg), barley (516 µg/kg), triticale (868 µg/kg), rye (1833 µg/kg), and oat (1951 µg/kg), related to their higher asparagine levels [43].

These results reinforce the relative advantage of Andean pseudocereals for thermally treated foods due to their significantly lower AA formation potential. Regarding f-AGEs, an increasing trend with heating time was observed in all varieties for both species (Figure 3B,D). However, quinoa showed higher values than cañihua in all varieties and times. Fluorescence intensity is related to the degree of protein modification by glycation [45]. Fluorescent AGEs peaked after 30 min of heating in quinoa “Cuchiwilla” while the rest of varieties peaked at 40 min, possibly associated with differences in free amino groups and their interactions with sugars and phenolic compounds.

Changes in AA and f-AGEs formation may be partially modulated by the phenolic composition of the flours, including phenolic acids and flavonoids (Supplementary Tables S1 and S2). Several studies have demonstrated that these compounds can interfere with Maillard reaction pathways through multiple complementary mechanisms. Phenolic compounds exhibit strong antioxidant activity, scavenging free radicals and limiting the oxidative degradation of sugars and Amadori intermediates. In addition, they are able to trap reactive dicarbonyl compounds such as glyoxal (GO), methylglyoxal (MGO), and 3-deoxyglucosone (3-DG), forming stable adducts that inhibit their conversion into AGEs. Polyphenols may also protect protein glycation sites by interacting with amino groups from lysine and arginine residues, thereby reducing their availability for glycation reactions. Furthermore, some phenolics can chelate transition metals (Fe^2+^ and Cu^2+^), reducing metal-catalyzed oxidative reactions associated with both glycation and AA formation [46,47,48]. Collectively, these mechanisms may explain the lower accumulation of AA and f-AGEs observed in cañihua compared with quinoa, particularly considering the differences in their phenolic profiles. This relationship is further supported by the PCA presented in Section 3.5, where phenolic compounds were associated with reduced levels of thermal contaminants and glycation markers. Although the present study did not directly quantify dicarbonyl trapping or radical scavenging during heating, the observed differences among varieties together with their phenolic profiles are consistent with previously described antiglycation and anti-AA formation mechanisms. Compounds such as gallic acid, protocatechuic acid, and flavonoids containing multiple hydroxyl groups have been reported as effective inhibitors of thermal contaminants and AGEs formation in complex food matrices.

### 3.5. Relationship Between Maillard Reaction Markers, AA, and f-AGEs

**Figure 4:**
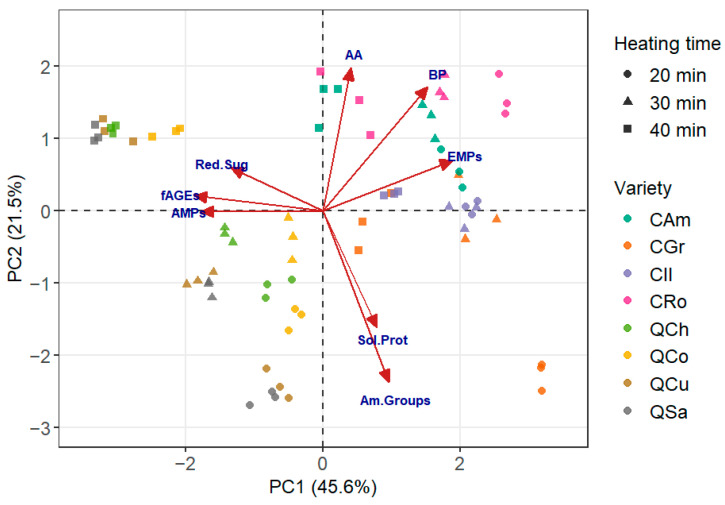
Principal component analysis (PCA) biplot for quinoa and cañihua samples subjected to different heating times at 185 °C.

To assess the relationship between key indicators of the MR, AA, and f-AGEs during heating, a non-supervised multivariate analysis (PCA) was performed. As shown in Figure 4 (biplot) the PC-1 explained 45.6% of the variability and separated the species, while the PC-2 explained 21.5% of the variability and separated the heating times. Together, these components explained 67.1% of the total variance of the dataset. The PC-3 explained 13.3% of the variance (80.4% cumulative variance, Supplementary Table S3, Supplementary Figures S2 and S3). For quinoa, all varieties treated for 20 and 30 min at 185 °C were grouped in the third quadrant, moving towards the second quadrant after 40 min heating. The quinoa samples treated for 40 min were correlated with reducing sugars, advanced Maillard products (AMPs) and f-AGEs, which underscores the influence of the formation of reducing sugars during heating on the formation of fluorescent products. For cañihua, most of the samples grouped in the first quadrant, and as heating time progressed, the samples moved towards the second quadrant.

The variables associated with cañihua samples were AA, brown pigments and early Maillard products. Interestingly, “Gris alfenica” started at the fourth quadrant and moved towards the center of the graph as heating progressed, suggesting that this variety accumulates average values of both MR markers, AA, and f-AGEs. This analysis remarks on the differential response of each species to thermal treatment and the necessity to take these differences into account when selecting the most appropriate processing time such that the formation of potentially harmful compounds is mitigated [4,43].

### 3.6. Model Systems Reveal the Role of Endogenous and Exogenous Phenolics on AA and f-AGEs Mitigation

**Figure 5:**
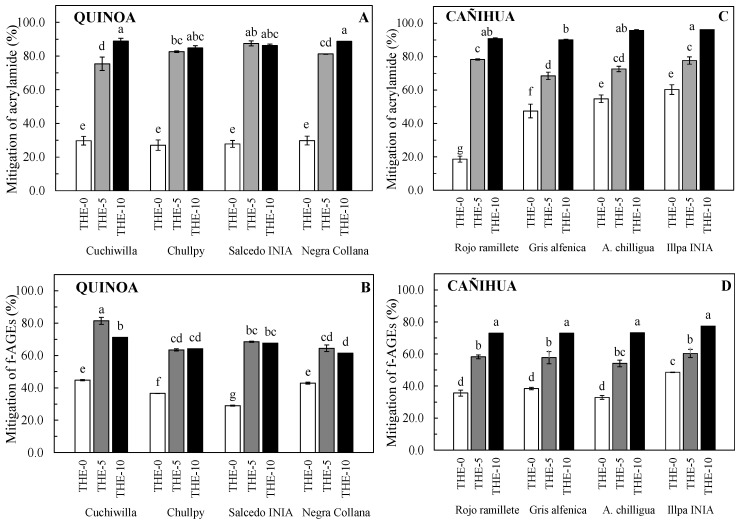
Effect of endogenous phenolics and exogenous phenolics (tara hydrolyzed extract) on the mitigation of Acrylamide (**A**,**C**) and f-AGEs (**B**,**D**). Bars show mitigation percentage with respect to pure Glc-Asn model system. Data are presented as means ± SD (n = 3). Different lowercase letters indicate significant differences (*p* < 0.05) determined by a two-way ANOVA (variety × exogenous phenolic concentration).

The equimolar model system consisting of key MR reactants Asn-Glc (0.1 M) was tested to better understand the effect of endogenous (flours with Asn and Glc concentrations adjusted to 0.1 M) and exogenous (THE at 0, 5 and 10 mg/mL) phenolics on AA and f-AGEs. Figure 5A–D show the mitigation percentage of each system with respect to the positive control (pure Asn and Glc). In the case of AA formation, for all quinoa varieties, the flour system showed mitigation levels around 25% with no significant differences among them (*p* > 0.05). However, there were varietal differences in AA formation for the cañihua flour system (*p* < 0.05) with “Amarillo chilligua” and “Illpa INIA” exhibiting the highest mitigation levels (over 50%), followed by “Gris alfenica” and “Rojo ramillete”, which exhibited lower inhibition percentages. These differences may be associated with endogenous phenolic compounds and antioxidant capacity among varieties and their interactions within the flour matrix.

During heating at 185 °C, reducing sugars may react competitively with free amino acids naturally present in quinoa and cañihua flours, including lysine, arginine, histidine, valine, and leucine, generating Maillard intermediates and melanoidins. This competitive consumption of carbonyl compounds may reduce the availability of reactive intermediates involved in AA formation. In parallel, endogenous phenolic compounds may interfere with Maillard reaction pathways through antioxidant and carbonyl-trapping mechanisms, further contributing to AA mitigation. Therefore, the inhibitory effect observed in flour systems cannot be explained exclusively by asparagine concentration, but rather by the complex interaction among free amino acids, reducing sugars, phenolic compounds, antioxidant capacity, and thermal reaction pathways occurring within the food matrix during heating.

The antioxidant capacity measured in the flour extracts (Supplementary Table S4) supports this hypothesis, suggesting that phenolic-rich systems may limit oxidative degradation reactions associated with the formation of reactive dicarbonyl intermediates. Jin et al. [49] demonstrated that gallic acid and protocatechuic acid reduce glucose oxidation and Amadori product degradation, thereby limiting the generation of 1,2-dicarbonyl compounds, which are key precursors of AGEs and AA. In the present study, gallic acid was identified in tara pod extract (Supplementary Table S5), whereas protocatechuic acid was detected in two quinoa varieties and at trace levels in all cañihua varieties (Supplementary Tables S1 and S2). These compositional differences may partially explain the higher mitigation observed in some cañihua systems.

The addition of tara pod hydrolyzed extract further enhanced AA mitigation in all varieties for both species. Inhibition was concentration-dependent, reaching higher values at the 10 mg GAE/mL concentration level (THE-10, Figure 5A,C) except for quinoa “Chullpy” and “Salcedo INIA” which showed no significant differences among THE concentrations. Indeed, this finding reinforces previous reports of hydrolyzed tara pod gallotannins as AA mitigators. Gallic acid is considered a potent anti-glycation agent because of its capacity to trap reactive carbonyls such as MGO and GO, key reactants for AA and AGEs formation, thus reducing the carbonyl pool available to react with amino acids [50]. Phenolic compounds present in pseudocereals (rutin) and tara pod (gallic acid) reduce AA formation in the Asn-Glc system by 49.4% and 47.7%, a higher mitigation rate than other known phenolics like chlorogenic acid, catechin and ferulic acid [17].

The inhibition levels observed in the present study are comparable to those previously reported for phenolic compounds in model asparagine–glucose systems. Previous studies have shown that rutin and gallic acid reduced AA formation by 49.4% and 47.7%, respectively, whereas chlorogenic acid and catechin exhibited lower inhibition rates (25.5% and 23.5%, respectively) under similar thermal conditions [51]. In the present study, cañihua flour systems supplemented with tara hydrolysate achieved mitigation levels above 50%, suggesting that the combined action of endogenous and exogenous polyphenols within the flour matrix may enhance inhibitory effects beyond those observed in simplified model systems. Similar inhibition ranges have been reported for gallic and protocatechuic acids in emulsion systems (up to 70%) [52] and for caffeoylquinic acids in glucose–asparagine systems (55–60%) [53], depending on matrix composition and phenolic concentration.

Fluorescent AGEs inhibition followed a similar pattern as AA. However, THE-10 exerted a greater inhibitory effect in cañihua, whereas no significant differences among THE concentrations were observed in quinoa (Figure 5B,D). Although there is no previous report on f-AGEs formation and mitigation in Asn-Glc systems to our knowledge, the observed reduction in fluorescence intensity may indicate the antiglycation activity of gallic acid and related polyphenols. Similar effects have been described for tea polyphenols [54] with over 60% of mitigation of f-AGEs in the gluten–glucose system, and inhibition levels up to 83.3% [55]. The inhibition percentages observed in the present study fall within the range reported for other cereal-based glycation systems enriched with polyphenols. However, the stronger inhibition observed in cañihua systems suggests that matrix composition and endogenous antioxidant compounds may play a critical role in modulating glycation pathways under thermal processing conditions. Although the present study did not directly quantify reactive dicarbonyl compounds or radical-scavenging reactions during heating, the combined evidence from antioxidant capacity, phenolic composition, and mitigation assays supports the proposed role of endogenous and exogenous polyphenols as inhibitors of AA and AGEs formation.

## 4. Conclusions

The results demonstrate that AA and f-AGEs formation in quinoa and cañihua flours is strongly influenced by varietal composition, particularly the levels of asparagine, reducing sugars, and phenolic compounds. Varieties exhibiting higher antioxidant capacity and enriched in quercetin and rutin accumulated lower levels of neo-formed contaminants and f-AGEs, suggesting a key inhibitory role of these flavonoids in Maillard reactions. Thermal treatment revealed distinct AA formation kinetics between species, with quinoa showing increased susceptibility to prolonged heating, whereas extended heating reduced AA levels in cañihua. Furthermore, the incorporation of exogenous phenolics from tara pods significantly mitigated AA and f-AGEs formation, achieving inhibition rates above 80%. Overall, the combined use of Andean pseudocereals and natural tara extracts represents a promising strategy for the development of functional foods with reduced levels of thermally induced contaminants.

## Figures and Tables

**Table 1:** Main sugars, asparagine, starch, total phenolics and antioxidant capacity.

Species	Variety	Glc (g/100 g DW)	Suc (g/100 g DW)	Asn (mg/100 g DW)	Starch (g/100 g DW)	TPC (mg GAE/g DW)	AC-ABTS (µmol TE/g DW)	AC-ORAC (µmol TE/g DW)
Quinoa	Cuchiwilla	0.17 ± 0.02 a	1.94 ± 0.09 b	11.33 ± 0.3 a	58.52 ± 0.36 b	1.47 ± 0.05 a	9.94 ± 0.11 a	24.68 ± 1.92 a
	Chullpy	0.19 ± 0.02 a	1.95 ± 0.06 b	7.54 ± 0.48 c	70.13 ± 3.27 a	1.15 ± 0.09 b	8.11 ± 0.09 b	17.17 ± 0.80 b
	Salcedo INIA	0.07 ± 0.01 b	2.17 ± 0.09 a	9.17 ± 0.58 b	74.81 ± 2.65 a	1.19 ± 0.02 b	7.20 ± 0.10 c	27.88 ± 1.32 a
	Negra Collana	0.09 ± 0.01 b	2.07 ± 0.07 ab	4.37 ± 0.13 d	62.05 ± 1.78 b	1.60 ± 0.03 a	10.04 ± 0.24 a	24.92 ± 0.75 a
Cañihua	Rojo ramillete	0.06 ± 0.00 b	2.16 ± 0.16 ab	11.69 ± 0.08 a	56.32 ± 3.69 a	2.60 ± 0.01 a	17.23 ± 0.89 b	82.44 ± 2.53 a
	Gris alfenica	0.05 ± 0.01 b	2.19 ± 0.07 a	9.32 ± 0.60 b	58.08 ± 2.29 a	1.81 ± 0.06 c	15.39 ± 0.19 b	69.75 ± 5.41 b
	Amarillo chilligua	0.10 ± 0.00 a	1.99 ± 0.08 b	3.13 ± 0.03 c	50.78 ± 4.21 a	2.50 ± 0.08 a	20.41 ± 1.17 a	78.89 ± 6.02 ab
	Illpa INIA	0.03 ± 0.00 c	2.32 ± 0.03 a	10.35 ± 0.84 ab	54.04 ± 3.79 a	2.14 ± 0.08 b	17.17 ± 1.00 b	77.68 ± 4.58 ab

Data are presented as means ± SD (n = 3). Different lowercase letters within the same column and species group indicate significant differences (*p* < 0.05).

## Data Availability

The original contributions presented in this study are included within the article and the Supplementary section. Further inquiries can be directed to the corresponding authors. During the preparation of this manuscript, the authors used Microsoft Copilot (on line version, Microsoft corporation) for the purposes of writing improvement. The authors have reviewed and edited the output and take full responsibility for the content of this publication.

## Supplementary Materials

The following supporting information can be downloaded at: https://www.mdpi.com/article/10.3390/foods15112010/s1. Supplementary Table S1. LC-PDA-QTOF MS profile and content of the phenolic compounds of the quinoa flour. Supplementary Table S2. LC-PDA-QTOF MS profile and content of the phenolic compounds of the cañihua flour. Supple-mentary Table S3. Total variance structure obtained by Principal Component Analysis. Explained and cumulative variance of each principal component. Supplementary Table S4. Total phenolic compounds content and antioxidant capacity (ABTS and ORAC) of the model system extracts, Supplementary Table S5. LC-PDA phenolic profile and content of the tara hydrolyzed extract, Supplementary Figure S1. Quinoa and cañihua grains. Q1: Negra Collana, Q2: Cuchiwilla, Q3: Salcedo INIA, Q4: Chullpy. C1: Rojo ramillete, C2: Gris alfenica, C3: Amarillo chilligua, C4: Illpa INIA, Supplementary Figure S2. Scree plot showing individual and cumulative explained variance obtained by PCA, Supplementary Figure S3. Principal Component Analysis (PCA) biplot showing PC1 and PC3 for quinoa and cañihua samples subjected to different heating times at 185 °C.

## Abbreviations

The following abbreviations are used in this manuscript:

AA	Acrylamide
MRPs	Maillard reaction products
NFCs	Neo-formed contaminant
THE	Tara Hydrolyzed Extract
