# Supplementary material for: Role of Endogenous and Exogenous Phenolic Compounds on the Formation of Acrylamide and Fluorescent Advanced Glycation End Products in Heated Quinoa and Cañihua Flours
Source: Foods. 2026 Jun 4;15(11):2010. doi: 10.3390/foods15112010 (PMC13257101; doi:10.3390/foods15112010)
Supplement: Supplementary file 1 [file foods-15-02010-s001.zip › foods-4318122-supplementary.pdf]

Supplementary Table S1. LC-PDA-QTOF MS profile and content of the phenolic compounds of the quinoa flour.

Peak N°	RT (min)	λ max (nm)	*m/z*	Error (ppm)	Formula [M-H]⁻	Fragments (relative abundance, %)	Annotation	Content (mg/g DW)†			
								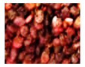 (1)	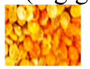 (2)	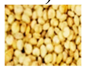 (3)	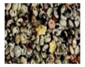 (4)
1	5.66	-	329.0877	1.2	C14H17O9	167.0347 (100), 329.0874 (36.29), 152.0112 (22.53), 168.0379 (9.47)	Vanillic acid derivative	nd	nd	nd	tr
2	5.86	259.6, 294.1	153.0191	2.0	C7H5O4	153.0191 (100), 109.0292 (7.07)	Protocatechuic acid*	13.56	nd	nd	6.99
3	6.71	-	341.0994	3.2	C15H17O9	179.0343 (100), 341.0926 (3.8), 161.0812 (3.7)	Caffeoyl hexoside	tr	nd	nd	tr
4	7.73	253.7, 291.7	137.0241	1.5	C7H5O3	137.0241 (100)	Hydroxybenzoic acid isomer	5.65	nd	nd	nd
5	8.13	-	137.0238	-0.7	C7H5O3	137.0238 (100)	4-Hydroxybenzoic acid*	tr	nd	nd	tr
6	8.17	-	355.1022	-2.0	C16H19O9	193.0495 (100), 178.0260 (15.37), 355.1022 (14.05), 194.0529 (11.73)	Glucosyl ferulate	nd	tr	tr	tr
7	8.34	-	153.0186	-1.3	C7H5O4	153.0186 (100), 109.0284 (3.73)	2,3-Hydroxybenzoic acid*	tr	nd	tr	tr
8	10.08	260.8, 291.7	167.0343	-0.6	C8H7O4	167.0343 (100), 152.0107 (30.67), 108.0210 (3.75), 123.0444 (3.67)	Vanilllic acid*	6.30	3.06	tr	tr
9	12.04	-	355.1028	-0.3	C16H19O9	193.0501 (100), 178.0267 (34.26), 355.1028 (28.18)	Glucosyl ferulate	nd	nd	tr	tr
10	13.57	-	459.1497	-1.3	C20H27O12	307.0851 (100), 459.1496 (56.32), 329.0668 (41.79), 163.0393 (35.15)	Unknown	nd	nd	nd	tr
11	15.02	308.4	163.0396	0.6	C9H7O3	163.0396 (100), 119.0497 (37.1)	p-Coumaric acid*	tr	tr	tr	tr
12	17.15	254.9, 354.4	755.2034	-0.1	C33H39O20	755.2032 (100), 300.0271 (24.66), 301.0321 (6.55), 271.0251 (1.18)	Quercetin glucoside (Quercetin-3-galactoside-6"-rhamnoside-3'"-rhamnoside)	135.28	139.98	36.34	235.75
13	17.37	254.9, 353.2	755.2032	-0.4	C33H39O20	755.2032 (100), 300.0273 (24.47), 301.0323 (6.50), 271.0245 (2.12)	Quercetin glucoside derivative	9.07	14.05	2.49	22.62

14	17.81	254.9, 353.2	741.1874	-0.5	C32H37O20	741.1874 (100), 300.0274 (25.55), 301.0329 (8.08), 271.0253 (1.40)	Quercetin glucoside derivative (Quercetin 3-(2G-xylosylrutinoside)	152.70	79.43	32.47	173.39
15	18.06	266.7, 314.3	741.1879	0.1	C32H37O20	741.1879 (100), 300.0275 (32.32), 301.0325 (9), 271.0247 (1.66)	Quercetin glucoside derivative	43.45	29.17	29.57	52.45
16	18.26	-	609.1447	-1.5	C27H29O16	609.1447 (100), 300.0262 (50.29), 301.0311 (14.91), 271.0233 (9.97), 255.0281 (5.16)	Quercetin glucoside derivative	nd	nd	nd	tr
17	18.33	-	279.0503	-0.7	C13H11O7	163.0393 (100), 279.0503 (74.97), 119.0495 (2.69)	p-Coumaric acid derivative (p-coumaroyl-malic acid)	tr	tr	tr	tr
18	18.39	265.5, 347.3	739.2089	0.4	C33H39O19	739.2089 (100), 284.0319 (18.20), 285.0380 (8.02), 255.0301 (1.60)	Kaempferol 3-O-(2,6-di-O-alpha-L-rhamnopyranosyl)-beta-D-galactopyranoside	127.03	125.06	350.22	135.04
19	18.46	264.3, 352.0	595.1302	0.5	C26H27O16	595.1302 (100), 300.0269 (48.41), 301.0321 (14.13), 271.0242 (13.89)	Quercetin derivative (3-arabinoglucosylquercetin)	3.05	2.05	5.28	9.32
20	18.52	265.5, 347.3	609.1100	1.3	C26H25O17	301.0344 (100), 609.1100 (70.11), 302.0377 (17.90), 300.0273 (5.75), 178.9980 (3.28)	Quercetin glucoside derivative	tr	tr	tr	tr
21	18.57	-	739.2084	-0.3	C33H39O19	739.2084 (100), 284.0322 (32.17), 285.0383 (12.69), 255.0297 (9.27)	Kaempferol glucoside derivative	nd	nd	tr	nd
22	18.61	-	595.1302	0.5	C26H27O16	595.1302 (100), 300.0277 (35.25), 271.0250 (16.69)	Arabinoglucosylquercetin derivative	tr	tr	nd	nd
23	18.61	-	595.1293	-1.0	C26H27O16	595.1293 (100), 300.0261 (41.84), 271.0235 (1.93)	Quercetin derivative (3-arabinoglucosylquercetin)	nd	nd	tr	tr
24	18.75	253.7, 354.4	769.2192	0.1	C34H41O20	769.2192 (100), 314.0426 (14.22), 315.0483 (5.89), 299.0190 (4.46)	Methoxyflavonol glucoside derivative	nd	9.39	nd	nd
25	18.73	-	609.1459	0.5	C27H29O16	609.1459 (100), 300.0274 (35.73), 301.0336 (16.81), 271.0247 (8.39), 167.0347 (2.65)	Quercetin glucoside derivative	tr	nd	nd	nd
26	18.82	265.5, 347.3	725.1926	-0.4	C32H37O19	725.1926 (100), 284.0319 (16.07), 285.0386 (8.87), 255.0296 (1.69)	Luteolin glucoside derivative	15.69	tr	50.43	9.07

27	18.89	-	769.2205	1.8	C34H41O20	769.2205 (100), 314.0426 (18.84), 315.0491 (11.09), 299.0191 (4.27), 300.0259 (3.03)	Methoxyflavonol glucoside derivative	tr	tr	nd	tr
28	18.93	355.6	609.1456	0.0	C27H29O16	609.1454 (100), 300.0271 (14.03)	Rutin*	2.38	nd	nd	nd
29	19.10	265.5, 347.3	593.1505	-0.2	C27H29O15	593.1505 (100), 284.0319 (17.47), 255.0296 (8.31)	Kaempferol glucoside derivative	nd	nd	3.18	nd
30	19.21	257.2, 358.0	477.0659	-2.1	C21H17O13	301.0341 (100), 477.0659 (91.95), 302.0378 (16.77), 178.9975 (7.36), 151.0026 (5.66)	Quercetin glucoronide	nd	tr	nd	26.35
31	19.56	265.5, 355.6	593.1496	-1.7	C27H29O15	593.1496 (100), 285.0405 (58.40), 495.2955 (50.73), 496.2992 (15.20)	Kaempferol glucoside derivative	nd	nd	12.86	nd
32	22.90	256.0, 371.3	301.0349	0.3	C15H9O7	301.0349 (100), 255.0657 (35.44), 302.0384 (17.82), 223.0945 (12.89), 178.9978 (8.96), 151.0028 (6.77)	Quercetin*	0.24	nd	nd	nd
**Total**								514.15	407.73	522.84	661.65

Values are presented as means (n = 2), nd: not detected, tr: trace amount (detected but not quantifiable).

*: Verified by previously injected authentic standard.

†(1) Cuchiwilla, (2) Chullpy, (3) Salcedo INIA, (4) Negra collana

Supplementary Table S2. LC-PDA-QTOF MS profile and content of the phenolic compounds of the cañihua flour.

Peak N°	RT (min)	λ max (nm)	*m/z*	Error (ppm)	Formula [M-H]$^-$	Fragments (relative abundance, %)	Annotation	Content (mg/g DW)†			
								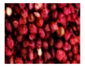 (1)	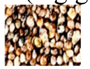 (2)	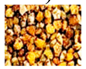 (3)	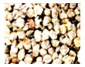 (4)
1	5.64	-	329.0871	-0.6	C14H17O9	167.0342 (100), 329.0871 (39.02), 168.0376 (9.36), 152.0107 (6.36), 218.1025 (5.76)	Vanillic acid derivative	tr	tr	tr	tr
2	5.86	-	153.0187	-0.7	C7H5O4	153.0187 (100), 109.0289 (19.98), 110.0328 (1.41)	Protocatechuic acid*	tr	tr	tr	tr
3	6.71	-	341.0883	2.9	C15H17O9	179.0345 (100), 341.0923 (39.79), 161.0813 (10.58)	Caffeoylglucopyranose (isomer I)	nd	tr	nd	nd
4	7.73	-	137.0239	0.0	C7H5O3	137.0239 (100)	Hydroxybenzoic acid isomer	tr	tr	tr	tr
5	8.13	-	137.0237	-1.5	C7H5O3	137.0237 (100)	4-Hydroxybenzoic acid*	nd	nd	tr	nd
6	8.17	232.4, 311.9	355.1026	-0.8	C16H19O9	193.0502 (100), 178.0267 (15.74), 355.1026 (14.62), 194.0536 (12.32)	Glucosyl ferulate (isomer I)	nd	tr	nd	nd
7	8.34	-	153.0189	0.7	C7H5O4	153.0189 (100), 108.0214 (7.92), 109.0288 (4.59)	2,3-Hydroxybenzoic acid*	tr	nd	tr	tr
8	8.84	-	341.0882	2.6	C15H17O9	179.0341 (100), 341.0889 (79.28), 180.0374 (8.17), 173.0809 (2.30)	Caffeoylglucopyranose (isomer II)	tr	tr	tr	tr
9	10.08	-	167.0345	0.6	C8H7O4	167.0345 (100), 152.0107 (28.62), 123.0453 (3.32), 108.0215 (2.79)	Vanillic acid*	tr	tr	tr	tr
10	12.04	-	355.1029	0.0	C16H19O9	193.0498 (100), 178.0262 (30.51), 215.0313 (30.05), 355.1029 (18.32)	Glucosyl ferulate (isomer II)	nd	nd	nd	tr
11	15.02	-	163.0397	1.2	C9H7O3	163.0397 (100), 119.0495 (38.90)	p-Coumaric acid*	tr	tr	tr	tr
12	17.15	254.9, 354.4	755.2035	0.0	C33H39O20	755.2035 (100), 300.0271 (27.35), 301.0323 (7.76), 271.0250 (2.02)	Quercetin glucoside (Quercetin-3-galactoside-6''-rhamnoside-3'''-rhamnoside)	541.02	45.29	154.89	153.70
13	17.37	256, 353.2	755.2031	-0.5	C33H39O20	755.2031 (100), 300.0270 (27.54), 301.0320 (7.02), 271.0244 (2.54)	Quercetin glucoside derivative	45.59	7.22	28.98	20.03

14	17.81	254.9, 352	741.1872	-0.8	C32H37O20	741.1872 (100), 300.0269 (22.71), 301.0323 (7.11), 271.0248 (1.61)	Quercetin glucoside derivative (Quercetin 3-(2G-xylosylrutinoside)	1,301.0	24.44	384.90	396.62
15	18.06	254.9, 354.4	741.1878	0.0	C32H37O20	741.1878 (100), 300.0283 (26.84), 301.0335 (7.34), 271.0263 (2.04), 255.0309 (1.00)	Quercetin glucoside derivative	426.59	74.84	159.07	180.21
16	18.39	265.5, 347.3	739.2089	0.4	C33H39O19	739.2089 (100), 284.0322 (17.05), 285.0382 (7.98), 255.0295 (1.85)	Kaempferol glucoside derivative (Kaempferol 3-(2G-rhamnosylrobinobioside)	80.26	16.40	19.94	22.22
17	18.61	256.0, 354.4	595.1318	3.2	C26H27O16	595.1318 (100), 300.0274 (60.59), 301.0322 (15.44), 271.0244 (13.85), 255.0294 (6.26)	Quercetin derivative (3-arabinoglucosylquercetin)	tr	tr	tr	tr
18	18.73	254.9, 354.4	609.1476	3.3	C27H29O16	609.1476 (100), 300.0273 (24.45), 301.0336 (11.27), 271.0253 (3.70), 255.0297 (1.96)	Quercetin glucoside derivative	656.46	117.35	196.88	222.64
19	18.90	-	769.219	-0.1	C34H41O20	769.2190 (100), 314.0423 (18.47), 315.0490 (10.63), 300.0266 (6.61), 299.0194 (4.79)	Methoxyflavonol glucoside derivative	tr	tr	tr	tr
20	18.97	254.9, 353.2	609.1459	0.5	C27H29O16	609.1473 (100), 300.0269 (19.20), 301.0339 (12.61), 271.0247 (3.38), 255.0293 (1.77)	Rutin*	707.14	91.23	249.47	231.36
21	19.21	253.7, 352	755.2039	0.5	C33H39O20	755.2048 (100), 314.0427 (18.48), 315.0490 (11.19), 299.0196 (1.97)	Methoxyflavonol glucoside derivative	56.39	18.22	24.79	34.86
22	20.05	254.9, 354.4	623.1611	-0.2	C28H31O16	623.1611 (100), 314.0427 (12.37), 315.0500 (12.30), 299.0198 (7.11), 300.0261 (3.46), 271.0245 (2.09)	Methoxyflavonol glucoside derivative	125.10	27.86	38.22	46.08
23	20.17	254.9, 354.4	623.1616	0.6	C28H31O16	623.1616 (100), 315.0504 (24.97), 314.0427 (6.24), 316.0536 (4.64), 299.0196 (2.50)	Narcissin (Isorhamnetin-3-O-rutinoside)	321.69	75.92	119.72	155.17
24	22.94	254.9, 354.4	301.0354	2.0	C15H9O7	301.0354 (100), 178.9983 (6.20), 151.0034 (5.02), 273.0403 (1.52)	Quercetin*	9.78	nd	30.58	nd
**Total**								4,271.0	498.8	1,407.4	1,462.9

Values are presented as means (n = 2), nd: not detected, tr: trace amount (detected but not quantifiable).

*: Verified by previously injected authentic standard.

†(1) Rojo ramillete, (2) Gris alfenica, (3) Amarillo chilligua, (4) Illpa INIA

Supplementary Table S3. Total variance structure obtained by Principal Component Analysis. Explained and cumulative variance of each principal component.

Principal Component	Explained Variance (%)	Cumulative Variance (%)
1	45.6	45.6
2	21.5	67.1
3	13.3	80.4
4	8.1	88.5
5	5.4	94.0
6	2.8	96.8
7	2.1	98.9
8	1.1	100.0

Supplementary Table S4. Total phenolic compounds content and antioxidant capacity (ABTS and ORAC) of the model system extracts.

Treatment	Quinoa				Cañihua			
	Cuchiwilla	Chullpy	Salcedo INIA	Negra collana	Rojo ramillete	Gris alfenica	A. chilligua	Illpa INIA
Phenolic compounds (mg GAE/mL)								
THE-0	0.29 ± 0.01c	0.23 ± 0.01c	0.23 ± 0.01c	0.30 ± 0.01c	0.24 ± 0.01c	0.24 ± 0.01c	0.22 ± 0.00c	0.19 ± 0.01c
THE-5	3.86 ± 0.15b	3.81 ± 0.10b	3.81 ± 0.08b	3.81 ± 0.10b	3.96 ± 0.13b	3.72 ± 0.17b	3.81 ± 0.07b	3.96 ± 0.12b
THE-10	7.52 ± 0.24a	7.61 ± 0.31a	7.60 ± 0.21a	7.53 ± 0.25a	7.97 ± 0.35a	7.70 ± 0.14a	7.69 ± 0.23a	7.59 ± 0.23a
ABTS antioxidant capacity (µmol TE/mL)								
THE-0	0.4 ± 0.0c	0.3 ± 0.0c	0.3 ± 0.0c	0.4 ± 0.0c	0.4 ± 0.0c	0.5 ± 0.0c	0.4 ± 0.0c	0.4 ± 0.0c
THE-5	132.6 ± 3.2b	122.5 ± 2.2b	125.2 ± 6.9b	126.0 ± 8.1b	125.5 ± 3.5b	129.3 ± 3.3b	127.8 ± 0.6b	129.4 ± 9.4b
THE-10	260.2 ± 3.4a	254.5 ± 3.8a	250.6 ± 3.5a	255.9 ± 4.2a	259.1 ± 3.9a	254.7 ± 1.4a	259.7 ± 4.9a	252.0 ± 8.4a
ORAC antioxidant capacity (µmol TE/mL)								
THE-0	0.9 ± 0.0d	0.6 ± 0.0d	1.0 ± 0.1d	0.9 ± 0.0d	3.1 ± 0.1c	2.7 ± 0.1c	2.7 ± 0.1c	3.0 ± 0.2c
THE-5	328.9 ± 22.7ab	342.1 ± 15.1ab	316.8 ± 15.3c	366.5 ± 32.1b	340.2 ± 3.9b	333.6 ± 15.6b	340.9 ± 6.8b	357.7 ± 20.8b
THE-10	698.3 ± 17.7a	677.5 ± 8.9a	661.0 ± 7.2a	668.5 ± 12.2a	676.5 ± 12.4a	678.1 ± 8.4a	688.7 ± 27.5a	690.3 ± 14.8a

Values presented as means ± SD (n = 3). Different letters within the same column indicate significant differences ($p < 0.05$).

Supplementary Table S5. LC-PDA phenolic profile and content of the tara hydrolyzed extract.

Peak N°	RT (min)	λ max (nm)	Annotation	Content (mg/g DW)
1	3.10	266.7	Gallic acid	833.06
2	10.27	272.7	Flavonol derivative	1.81
3	19.75	253.7, 367.7	Flavone derivative	tr
4	20.42	282.2	Hydroxybenzoic acid derivative	0.76
5	24.49	276.2	Hydroxybenzoic acid derivative	0.46
6	29.62	276.2	Hydroxybenzoic acid derivative	tr
Total				836.10

Values are presented as means (n = 2), tr: trace amount (detected but not quantifiable).

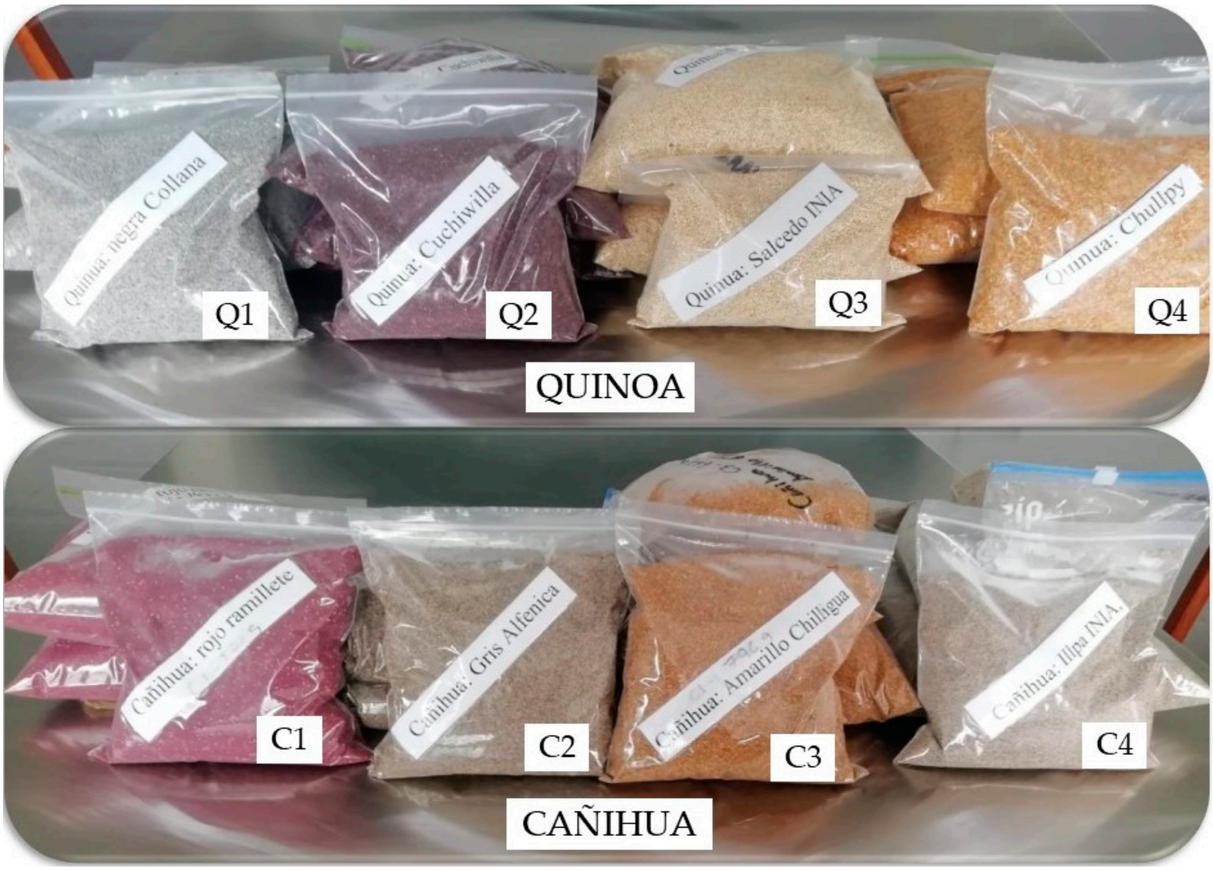

Supplementary Figure S1. Quinoa and cañihua grains. Q1: Negra collana, Q2: Cuchiwilla, Q3: Salcedo INIA, Q4: Chullpy. C1: Rojo ramillete, C2: Gris alfenica, C3: Amarillo chilligua, C4: Illpa INIA.

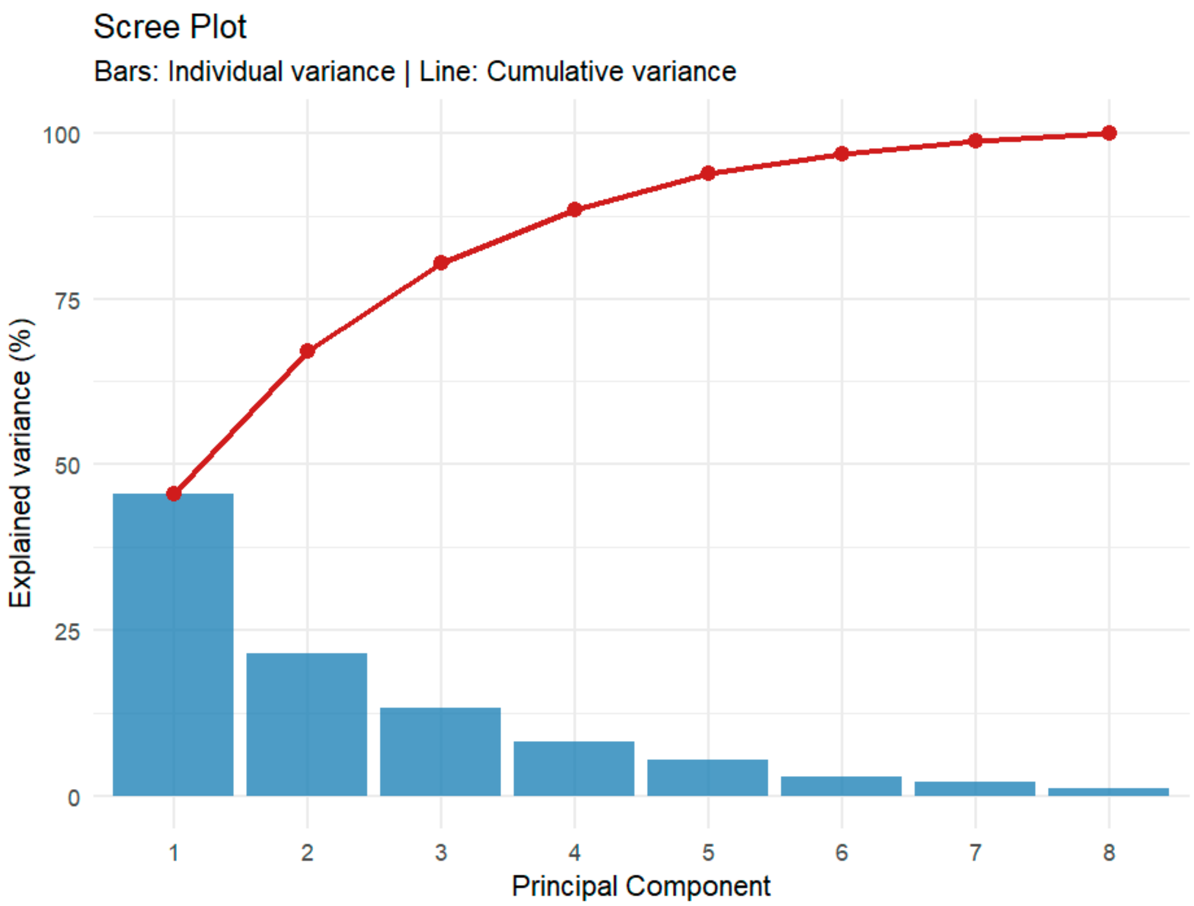

Supplementary Figure S2. Scree plot showing individual and cumulative explained variance obtained by PCA.

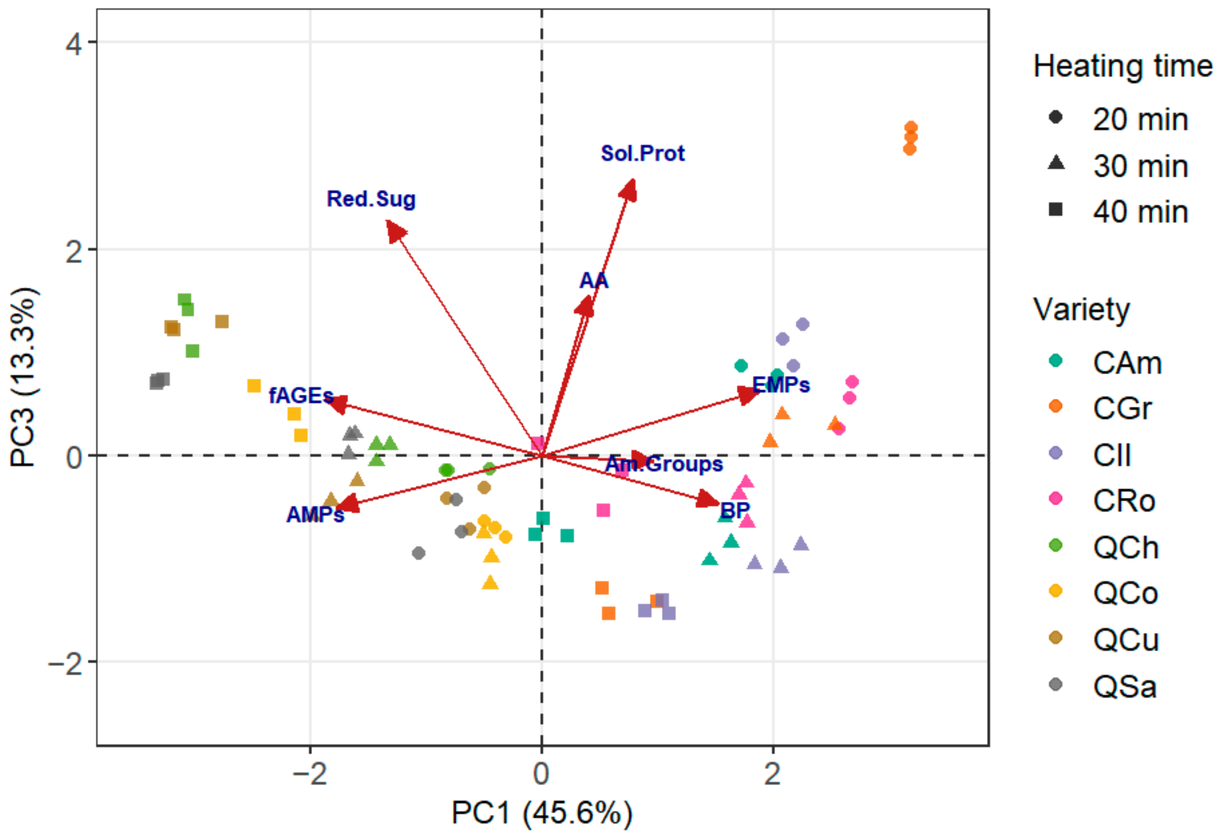

Supplementary Figure S3. Principal Component Analysis (PCA) biplot showing PC1 and PC3 for quinoa and cañihua samples subjected to different heating times at 185 °C.
